# Depression, anxiety and associated factors among housemaids working in Addis Ababa Ethiopia

**DOI:** 10.1186/s12888-020-02638-5

**Published:** 2020-05-13

**Authors:** Alem K. Ejigu, Zahra R. Seraj, Mahlet W. Gebrelibanos, Tolesa F. Jilcha, Yodit H. Bezabih

**Affiliations:** 1 Postcode 185093 Addis Ababa, Ethiopia; 2 Postcode 1971 Addis Ababa, Ethiopia

**Keywords:** Depression, Anxiety, Housemaid, Ethiopia

## Abstract

**Background:**

Housemaids are part of women with low socioeconomic status and most of them are migrant from rural to central part of Ethiopia, less educated, either with poor, separated, single or divorced family and/or dead parents. Housemaid may experience problems like depression and anxiety more than other groups of women. Therefore, the aim of this study was to assess the magnitude and determinants of those problems among housemaids.

**Objective:**

To assess the magnitude and determinants of depression & anxiety among housemaids in Addis Ababa; Ethiopia; 2018.

**Methods:**

Community based cross-sectional study was conducted from May 1 to August 30, 2018 among housemaids working in Addis Ababa, Ethiopia. Multistage sampling technique was applied with a total of 826 samples. Quantitative data was employed by using structured questionnaires. The collected data was coded, entered in to Epi-Info version 7 and analyzed by using SPSS version 20. Descriptive, analytical statistical procedures; bivariate and multivariate binary logistic regressions with odds ratios and 95% confidence interval was employed. The statistical significance was declared at p value < 0.05.

**Results:**

This study enrolled 862 participants with response rate 99.5%, 99.5% refers to the number of people who actually completed the interview. The result showed prevalence of depression and anxiety among housemaids 27.5% and 32.3% respectively. Among all participants 44.6% (95% CI= 41.0 – 47.9) have mild, 18.5% (95% CI= 15.7 – 21.2) have moderate and 6.1% (95% CI= 4.5 – 7.8) have severe form of comorbid anxiety with depression. Depression (44.9%) and anxiety (41.9%) found more prevalent among the age group 16 to 20. In this study history of parental divorce, participant’s divorce, physical violence and sexual violence are positively associated. Other factors; being less educated and living with relatives; were associated negatively.

**Conclusion:**

The prevalence of Depression and Anxiety is found high among housemaid; its prevalence is more among age group 16 to 20 than other age groups. Violence, participant’s divorce, history of parental divorce and contraceptive use has positively associated with depression and anxiety.

## Background

### Statement of the problem

Depression and Anxiety are common mental health problems among women worldwide [1–4]. In case of anxiety and depression; the greater vulnerability of women varies with the age: before mid-puberty, boys are more likely than girls to anxious or depressed, while between 15 and 19 years, the prevalence of depression is doubled in girls [2, 5]. This trend persists until 54 years [3]. Depression is part of mental illness which is more prevalent among women and women in socioeconomic problems are more risky when compare with women in better socioeconomic status [6].

Housemaids are a relatively homogenous immigrant subgroup in terms of their gender, socio-cultural, educational and occupational background. Psychiatric morbidity among housemaids is two to five times higher than the native female population [7–9].

Housemaid the most common occupation in the female labor force which can generate mental suffering such as depressive and anxiety symptoms [10].

Poverty and hunger force many women to migrate as a survival strategy. In many countries, women migrants working in domestic service or factories are at high risk of experiencing abuse by employers including confinement, slave-like conditions, and physical and sexual assault in turn that may lead them to develop depressive or anxiety symptoms [7, 11].

Housemaids are part of community who leave their primary living place or their families because of challenging life events that obligate them to search other alternatives to survive or for perceived better life. The majority of housemaids are women, poor and immigrants with minimal education [7, 9, 12].

In our country context responsibilities of housemaids vary and generally include cleaning & arranging the house, compartment & households; giving care for children & entire families; cooking & serving food for family members; in some context shopping & managing the house at all. The specific tasks vary in each household’s standard, family size and each employer’s perception. Performing all those activities may lead them to stress and stress related problems [1, 13, 14].

Although there are many researches done; in the world including Ethiopia; on depression and anxiety among women, there is a serious lack of information on the magnitude, as well as factors associated with depression and anxiety among housemaid. This study was therefore aim at evaluating the magnitude and determinants of depression and anxiety among housemaids in Addis Ababa, Ethiopia.

### Objectives

#### General objective:

To assess magnitude and determinants of depression and anxiety among housemaids in Addis Ababa, Ethiopia; 2018

#### Specific objectives:

- ❖ To assess magnitude of depression among housemaids in Addis Ababa, Ethiopia; 2018
- ❖ To assess magnitude of anxiety among housemaids in Addis Ababa, Ethiopia; 2018
- ❖ To assess determinants of depression among housemaids in Addis Ababa, Ethiopia; 2018
- ❖ To assess determinants of anxiety among housemaids in Addis Ababa, Ethiopia; 2018

## Methods

### Study Design and Period

Community based Cross-sectional study was conducted from May 1 to August 30, 2018 in Addis Ababa.

### Study Setting

Addis Ababa is Ethiopian capital city which contained different groups of population in different socioeconomic status. The city has a group of population from entire parts of the country. Among them woman are part of population migrate to capital city due to different reasons like occupation, relationship, education and other socioeconomic problems. Occupational migrants include woman who engage in different labor works to survive and to get perceived better life. Shelter is among major security and safety problem for those migrant women. They struggle to solve such housing problems in different approaches like forming large group and sharing poor setting house, renting daily paid settings for night, living on the street, possibly depend on relatives and in most cases of women in labor worker; they employee as housemaid to solve those major housing problems. Housemaids are among member of community in Addis Ababa population. Majority of them are migrants form entire (East, West, South and Northern) parts of the country for perceived better life with limited knowledge about urbanization, low socioeconomic status and with major communication barriers. Situations in the capital town like infrastructures, culture, values and norms are stranger to them. Also they start working in the city without safety, security, training and orientation about the new environment, job and life style.

### Source and Study Population

#### Source Population

All housemaids working in Addis Ababa, Ethiopia

#### Study Population

Our study population was all housemaids working in Addis Ababa who were available at the time of data collection in selected households.

### Eligibility Criteria

Housemaids who have work experience more than six consecutive months back from data collection date were included in this study.

Non residential workers (not living together with employer) and housemaids who were previously diagnosed mental illness were excluded in this study.

### Sample Size and Sampling Procedures

#### Sample Size Determination

The minimum number of sample required for this study was estimated by using Single population proportion formula considering the following assumptions

$$ n=\frac{{\left(\frac{Z\alpha}{2}\right)}^2p\left(1-P\right)}{d^2} $$

Where

n = minimum sample size required for the study

Z= standard normal distribution (Z=1.96) with confidence interval of 95% and ⍺=0.05

d= margin of sample error tolerated, (d) =5%=0.05

P= Proportion the sample size was determine for each of response variables and the maximum sample size was take

Considering non response rate of 15%, the total sample size for the study was 826

#### Sampling Techniques

A multistage sampling technique was used and 3 sub cities selected from 10 sub cities. Then 3 Woredas selected from each sub cities by using simple random sampling method. The sample size was distributed to each selected Woredas proportionally to its household size. Computer generated lottery method was use to select household from each woreda, if there is no housemaid in selected household next or previous household was checked. In case of more than one housemaid at a household one selected randomly.

### Instruments used for data collection and data collection procedures

#### Instruments Used for Data Collection

In this study, to assess Socio-demographic information, clinical data, depression, anxiety, factors associated with depression and to assess factors associated with anxiety we were using interviewer administered questionnaire. The questionnaire has five parts: 1^st^ part contains Sociodemographic data, 2^nd^ part contains depression and anxiety assessment tool, 3^rd^ part includes questions to assess violence (Physical, Emotional and sexual violence), 4^th^ part to assess presence of stressful life events and the last part contain semi structured questions to assess clinical data of participants.

Depression and anxiety were measured by using PHQ -4. It is four items assessment tools which have separate parts for depression and for anxiety. Two items for depression (the PHQ-2 has a sensitivity of 83% & specificity of 90%) and two items for anxiety (GAD-2 has sensitivity 88% & specificity 81%–83%). Also PHQ -4 has potency to measure occurrence of both cases at a person. All the 4 items should answered on a four point (Likert type) scale from “not at all” through “several days” and “more than half the days” to “nearly every day” [15]. To assess violence, WHO violence assessment tool was used. It has three parts: sexual violence which has six questions, emotional violence and physical violence which has three questions for each. Presence of one ‘Yes’ in each category is enough to say possible domestic violence [16]. Daily Stress Full Life Events Measurement Scale (DSLEMS) to assess presence of Stressful Life Events and to obtain socio-demographic and clinical data a detailed self-designed semi structured questionnaire was administered to consented study participants.

#### Data collection Procedure

Quantitative data was employed to collect information. Amharic Version of structured and semi structured interviewer administered questionnaire was used to collect necessary data from study participants. The data was collected by 9 female health extension workers for 18 weekends.

#### Data Quality Management

To assure the quality of the data high emphasis was give in designing data collection instruments for its simplicity. Prior to data collection training was give for data collectors. The questioner was pretested before the actual data collection activities and necessary modification was done. The collected data was reviewed and checked for its completeness and consistency.

### Definition of Variables

In this study depression and anxiety are dependent variables and socio demographic factors (age, educational level, marital status, salary, service year and family status), clinical factors **(**medical illness, family history of mental illness, Medications) and psychological factors (substance use, stress, violence) are independent variables.

**Anxiety**: it is explained by score on PHQ 4: a cut of point 3 and more on the 0-to-6 –point GAD–2 scale determine as anxiety [15].

**Depression**: score on PHQ 4: a cut of point 3 and more on the 0-to-6 point PHQ–2 score determine as depression [15].

**Depression with Anxiety**: PHQ–4 scores were operationally categorized as mild, moderate and severe forms of comorbid depression with anxiety. ‘mild’ for PHQ -4 scores 3 to 5, ‘moderate’ for PHQ -4 scores 6 to 8 and ‘severe’ for PHQ -4 scores 9 to 12 [15].

**House head**: in this study it includes all independent house heads inside each formally numbered household

**Housemaids**: Females who are working in household either non residential (not living with employer) or residential (living with employer) system.

### Data Analysis

Data was coded and entered into Epi-Info version 7 for cleaning, storing and recording. Then the data imported to computer software Statistical Package for Social Sciences version 20 (SPSS-20). Descriptive statistical procedures were utilized to estimate the prevalence of depression and anxiety among housemaids. Bivariate and multivariable logistic regression analyses were conducted to identify associated factors for depression and anxiety among housemaids. The strength of the associated factors was presented by odds ratio with 95% CI. The independent variables that fulfill p-value less than 0.2 were tolerated and exported to multivariable logistic regression. P-values less than 0.05 were considered as statistically significance.

### Dissemination and Utilization of results

The result of this cross sectional study will consider disseminating and allowed to utilize the following organizations.

- ➢ To Amanuel Mental Specialized Hospital
- ➢ To Federal minster of health
- ➢ To Addis Ababa health bureau
- ➢ Ethiopia Ministry of Women, Children and Youth Affairs
- ➢ To publish the research in scientific journals and to present it in different scientific conference.

## Results

### Description of Socio Demographic Characteristics of Respondents

This study enrolled 862 participants with response rate 99.5%, 99.5% is the number of people who actually completed the interview. Average age of housemaids was 20 ± 4.53; range 15 to 46 years. Most (47.1%) of the study participants were in the age group 16 to 20, 27.5% of participants has work experience of 2 to 3 years. Majority (63.3%) of participants were at primary level of Education, 60.9% were orthodox in religion. Majority (56.9%) of respondent’s salary is in range 501 to 1000. Around 70% of participants were single in relationship, 37.2% visit their families more than a yearly bases, 25.7% of participants lost one of their parents, 65.9% of participants families not found nearly (Table 1).

**Table 1:** Socio-demographic characteristics of housemaids: Addis Ababa, Ethiopia - 2018

Variables	Results: ***n***=826
	Frequency (%)
**Age**	
15	29 (3.50)
16 – 20	389 (47.10)
21 – 25	85 (10.30)
>25	323 (39.10)
**Service Years**	
6 months	143 (17.30)
1 to 2 years	191 (23.10)
2 to 3 years	228 (27.60)
3 to 5 years	69 (8.40)
>5 years	195 (23.60)
**Educational status**	
No formal education	127 (15.40)
Primary education	523 (63.30)
Secondary education	163 (19.70)
Higher education	13 (1.60)
**Religion**:	
Orthodox	503 (60.90)
Muslim	112 (13.60)
Protestant	209 (25.30)
Catholic	2 (0.20)
**Salary:**	
**<**500	36 (4.40)
501 -1000	470 (56.90)
1001 – 1500	174 (21.10)
1501 – 1200	98 (11.90)
>2000	48 (5.80)
**Marital Status:**	
Single	660 (79.90)
Separated	86 (10.40)
Divorced	66 (8.00)
Widowed	6 (0.70)
Married	8 (1)
**Family Visit:**	
<monthly	125 (15.10)
Monthly	174 (21.10)
More than monthly	185 (22.40)
Yearly or more	307 (37.20)
Living with employer	34 (4.10)
Missed	1

### Description of psychological factors among housemaids

Among 862 participants, if problem happened 51.7% have difficulties to get family support. Prevalence of lifetime physical and sexual violence was 22.8 & 12.5% respectively. Also 6.2% of participants had sexual violence at the age under 15. Majority (73%) of participants has domestic violence. Emotional violence was the most (64.30) prevalent type of violence when compared with physical and sexual violence (Table 2).

**Table 2:** Psychosocial characteristics of housemaids: Addis Ababa, Ethiopia - 2018

Variables	Results: ***n***=826	
	Yes	No
Life time physical violence	188 (22.8)	638 (77.20)
Hard to get family support	427 (51.70)	399 (48.30)
Life time sexual violence	103 (12.50)	723 (87.50)
Under 15 Sexual Violence	51 (6.20)	775 (93.80)
Daily Stressful Life Events	217 (26.30)	609 (73.70)
Domestic violence:	603 (73.00)	223 (27.00
Emotional	531 (64.30)	295 (35.70)
Physical	378 (45.80)	448 (54.20)
Sexual	169 (20.50)	657 (79.50)
Substance use		
Khat	14 (1.70)	812 (98.30)
Alcohol	32 (3.90)	794 (96.10)
Cigarette	8(1.00)	818 (99.00)

### Description of clinical factors among housemaids

Among all participants 6.2% have known medical illness and among them 41.18 take medication for medical illness. Also 14.5% of participants use contraceptive (Table 3).

**Table 3:** Clinical characteristics of housemaids: Addis Ababa, Ethiopia - 2018

Variables	Results: *n*=826	
	Yes	No
Previously known medical cases	51 (6.20)	775 (93.80)
Medication for known case: (*n*=51)	21 (41.18)	30(58.82)
Contraceptive use	120 (14.50)	706 (85.50)

### Prevalence of depression and anxiety among housemaid

#### Prevalence of Depression among housemaid

In this study prevalence of depression found 27.5% (95% CI= 24.3 – 30.40). It is more (44.9%) prevalent among the age group 16 to 20 year.

#### Prevalence of Anxiety among housemaid

Anxiety found more prevalent than depression it counts 32.3% (95% CI= 29.3 - 35.4). It accounts equal amount among the age groups 16 to 20 (13.6%) and age group above 25 years (13.6%).

#### Prevalence of Comorbid Depression with Anxiety among Housemaid

Among all participants 44.6% (95% CI= 41.0 – 47.9) have mild, 18.5% (95% CI= 15.7 – 21.2) have moderate and 6.1% (95% CI= 4.5 – 7.8) have severe form of comorbid Depression with Anxiety.

### Factors Associate with Anxiety and Depression among Housemaids

#### Factors associated with Depression

The binary logistic (crude analysis) was done on socio-demographic (Age, marital status, educational status, service year, salary, family visit, Family support and parent’s relationship status), clinical (contraceptive use); psychosocial factors (alcohol use, khat use, stress full life events, life time physical and sexual violence, under 15 sexual violence and domestic violence, ) were found to be p-value of less than 0.2 in Univariate logistic regression and taken in to multivariable logistic regression. The variable group expected to be protective against depression is treated as the reference group (Table 4).

**Table 4:** Factors which has association with depression on binary logistic regression: Addis Ababa, Ethiopia- 2018

Variables	Results: ***n***=826			
	Depression		COR (CI 95%)	AOR (CI 95%)
	Yes	No		
**Educational Status**				
No Formal Education	44 (19.40)	83 (13.90)	.618 (.196 -1.953)	0.28(0.08 - 0.98)^*^
Primary Level	134 (59.00)	309 (64.90)	.402 (.133 – 1.217)^*^	0.24 (0.07 - 0.82)^*^
Secondary Level Education	43 (18.90)	120 (20.00)	.418 (.1333 – 1.313)^*^	0.25 (0.07 – 0.87)^*^
College Level Education	6 (2.60)	7 (1.20)	1.00	1.00
**Relationship status**				
Single	163 (71.80)	497 (83.00)	1.00	1.00
Separated	25 (11.00)	61 (10.20)	1.25 (0.76 – 2.06)	1.27 (0.75 – 2.19)
Divorced	33 (14.50)	33 (5.50)	3.05 (1.82 – 5.10)^***^	2.62 (1.46 – 4.71)^**^
Widowed	4 (1.80)	2 (0.30)	6.10 (1.102 – 33.60)^*^	1.54 (0.25 -9.64)
Married	2 (0.90)	6 (1.00)	1.02 (0.203 – 5.09)	1.15 (0.22 – 6.08)
**Vacation**				
Less than monthly	32 (14.10)	93 (15.50)	1.00	1.00
Monthly	51 (22.50)	123 (20.50)	1.23 (0.72 – 2.02)	0.83 (0.46 – 1.47)
Monthly to yearly	36 (15.90)	149 (24.90)	0.70 (0.41 – 1.21)	0.52 (0.29- 0.95)^*^
More than Yearly	92 (40.50)	215 (35.90)	1.244 (0.78 – 1.99)	0.83 (0.49 – 1.40)
Living with employer	16 (7.00)	19 (3.20)	2.45 (1.13 – 5.32)^*^	1.86 (0.79 – 4.38)
**Parent R. condition**				
Living together	87 (38.30)	321 (53.60)	1.00	1.00
Separated	22 (9.70)	60 (10.00)	1.35 (0.77 – 2.33)	1.38 (0.76 – 2.52)
Divorced	21 (9.30)	32 (5.30)	2.42 (1.33 – 4.41)^**^	2.10 (1.08 -4.10)^*^
Mother/ father lost	77 (33.90)	135 (22.50)	2.10 (1.46 – 3.04)^***^	1.98 (1.31 – 2.99)^**^
Both parents lost	20 (8.80)	51 (8.50)	1.45 (0.82 – 2.55)	1.22 (0.64 – 2.34)
**Daily Stressful Life E**				
Yes	76 (33.50)	141 (23.50)	1.99 (1.45- 2.72)***	1.31 (0.89 – 1.9 1)
No	151 (66.50)	458 (76.50)	1.00	
**<15 Sexual V.**				
Yes	24 (10.60)	27 (4.50)	2.50 (1.41 – 4.44)**	1.81 (0.84 – 3.88)
No	203 (89.40)	572 (95.50)	1.00	1.00
**Physical violence**				
Yes	131 (57.70)	247 (41.20)	1.94 (1.43 – 2.65)^***^	1.63 (1.13 -2.37)^*^
No	96 (42.30)	352 (58.80)	1.00	1.00
**Emotional Violence**				
Yes	168 (74.00)	363 (60.60)	1.85 (1.32 – 2.59)***	1.32 (0.87 – 2.00)
No	59 (26.00)	236 (39.40)	1.00	1.00
**Adolescence Sexual V.**				
Yes	69 (30.40)	100 (16.70)	2.18 (1.53 – 3.11)^***^	1.59 (1.01 – 2.52)^*^
No	158 (69.60)	499 (83.30)	1.00	1.00
**Contraceptive Use**				
Yes	26 (11.50)	94 (15.70)	0.69 (0.44 – 1.10)^***^	2.19 (1.31 – 3.67)^**^
No	201 (88.50)	505 (84.30)	1.00	1.00

*COR* Crude Odds Ratio, *AOR* Adjusted Odds Ratio

N.B. *=*p*-value<0.05, **p-value<0.01, ***p-value<0.001

The odds of developing depression is increased 2.62; (AOR=2.62, 95% CI= 1.46 – 4.71); times among divorced participants when compared with the single one.

The odds of developing depression is decreased by 48%; (AOR=0.52, 95%CI=0.29- 0.95); among participants who visit families in the period monthly to yearly when compared with those visit parents less than monthly.

The odds of developing depression is 2.10; (AOR=2.1, 95% CI=1.08 – 4.10); times increased among participants whose parents are divorced and it increased 1.98; (AOR=1.98, 95%ci= 1.31 to 2.99) times among participants who lost one of their parents when compared with participants whose parents are living together.

The odds of developing depression is 1.63; (AOR=1.63, 95% CI =1.13 – 2.37); times increased among housemaid who had physical violence.

The risk of developing depression is 1.59; (AOR=1.59, 95%CI=1.01 – 2.52); times higher among participants who had sexual violence.

The odds of developing depression is 2.19; (AOR=2.19, 95%CI= 1.31 – 3.67); times increased among housemaids who are using contraceptive than who are not using contraceptive.

However, in this study there is no significant association identified between depression and educational status, service year, DSLE, sexual violence (under 15 & life time) and emotional violence.

#### Factors associated with anxiety:

The binary logistic (crude analysis) was done on socio-demographic (Age, marital status, educational status, service year, salary, family visit and parent’s relationship status), clinical (medical condition, contraceptive use); psychosocial factors (alcohol use, khat use, stress full life events, life time physical and sexual violence, under 15 sexual violence and domestic violence, ) were found to be p-value of less than 0.2 in Univariate logistic regression and taken in to multivariable logistic regression. The variable group expected to be protective against anxiety is treated as the reference group (Table 5).

**Table 5:** Factors which has association with anxiety on binary logistic regression: Addis Ababa, Ethiopia- 2018

Variables	Results: ***n***=826			
	Anxiety		COR (CI 95%)	AOR (CI 95%)
	Yes (%)	No (%)		
**Age**				
<15	13 (4.90)	16 (2.90)	1.53 (0.71 – 3.29)	1.89 (0.79 – 4.53)
16 to 20	112 (41.90)	227 (49.60)	0.76 (0.55 – 1.05)*	0.83 (0.56 – 1.21)
21 to 25	30 (11.20)	55 (9.80)	1.03 (0.62 -1.69)	0.71 (0.38 -1.33)
>25	112 (41.90)	211 (37.70)	1.00	1.00
**Educational status:**				
No formal Education	44 (19.40)	83 (13.90)	0.62 (1.96 – 1.95)	0.28 (0.08 – 0.98)*
Primary level	134 (59.00)	309 (64.90)	0.40 (0.13 – 1.22)*	0.24 (0.07 – 0.82)*
Secondary level	43 (18.90)	120 (20.00)	.042 (0.13 – 1.13)*	0.25 (0.07 – 0.87)*
College level	6 (2.60)	7 (1.20)	1.00	1.00
**Salary**				
<500	4 (1.50)	32 (5.70)	0.19 (0.58 – 0.63)**	0.25 (0.02 – 2.72)
501 – 1000	161 (60.30)	309 (55.30)	0.79 (0.43 – 1.46)	1.45 (0.25 – 8.21)
1001 – 1500	48 (18.00)	126 (22.50)	0.58 (0.29 – 1.13)*	0.93 (0.21 – 4.06)
1501 – 2000	35 (13.10)	63 (11.30)	0.85 (0.42 – 1.73)	1.31 (0.42 – 4.13)
>2000	19 (7.10)	29 (5.20)	1.00	1.00
**Relatives Distant (how far if your families?)**				
Nearest as possible to visit	167 (62.50)	377 (67.40)	1.00	1.00
Not found nearly	2 (0.70)	7 (1.30)	0.36 (0.10 – 1.30)	2.63 (0.62 – 11.11)
Living with them	92 (34.50)	171 (30.60)	0.29 (0.08 – 1.06)*	0.62 (0.42 – 0.92)*
I don’t know where they are	6 (2.20)	4 (0.70)	0.19 (0.02 – 1.43)	3.30 (0.69 – 15.78)
**Relationship status**				
Single	200 (74.90)	460 (82.30)	1.00	1.00
Separated	26 (9.70)	60 (10.70)	0.99 (0.61 – 1.36)	1.20 (0.67 – 2.15)
Divorced	35 (13.10)	31 (5.50)	2.59 (1.56 – 4.33)***	3.92 (1.99 – 7.69)***
Widowed	4 (1.50)	2 (0.4)	4.60 (0.84 – 25.32)*	1.56 (0.20 – 12.22)
Married	2 (0.70)	6 (1.10)	0.77 (0.15 – 3.83)	1.19 (0.22 – 6.61)
**Vacation (**how frequent do you visit your families?)				
Less than monthly	26 (9.70)	99 (17.70)	1.00	1.00
Monthly	56 (21.00)	118 (21.10)	1.891 (1.06 – 3.09)*	1.07 (0.59 – 1.96)
Monthly to yearly	56 (21.00)	129 (23.10)	1.65 (0.97 – 2.82)*	0.59 (0.32 – 1.12)
More than Yearly	117 (43.8)	190 (34.00)	2.34 (1.44 - 3.82)**	1.15 (0.65 – 2.04)
Living with employer	12 (4.50)	23 (4.10)	1.99 (0.87 – 0.51)	2.63 (1.06 – 6.56)*
**Parent condition (Parent Relationship status)**				
Living together	113 (42.30)	295 (52.80)	1.00	1.00
Separated	31 (11.60)	51 (9.10)	1.59 (0.97 – 2.61)*	1.12 (0.59 – 2.10)
Divorced	25 (9.40)	28 (5.00)	2.33 (1.30 – 4.19)**	2.45 (1.23 – 4.89)*
Mother/ father lost	74 (27.70)	138 (24.70)	1.40 (0.98 – 1.99)*	1.98 (1.30 -3.02)**
Both parents lost	24 (9.00)	47 (8.40)	1.33 (0.78 – 2.28)	1.13 (0.58 -2.19)**
**Parent support: If problems happen, how hard is getting family support?**				
Yes	106 (41.20)	28.90(51.70)	0.65 (0.49 -0.89)**	1.67 (1.16 – 2.42)**
No	157 (58.80)	270 (48.30)	1.00	1.00
**Physical Violence: while working as house maid**				
Yes	153 (57.30)	225 (40.30)	1.99 (1.48 – 2.68)**	1.59 (1.09 – 2.33)*
No	114 (42.70)	334 (59.70)	1.00	1.00
**Sexual Violence: while working as house maid**				
Yes	93 (34.80)	76 (13.60)	3.39 (2.39 – 4.82)***	1.72 (1.07 – 2.78)*
No	147 (65.20)	483 (86.40)	1.00	1.00
**DSLE:**				
Yes	96 (36.00)	121 (21.60)	2.03 (1.47 – 2.80)***	1.51 (1.04 – 2.19)*
No	171 (64.00)	438 (78.40)	1.00	1.00
**Contraceptive Use**				
Yes	106 (41.20)	289 (51.70)	0.73 (0.47 – 1.12)*	2.03 (1.18 – 3.50)*
No	157 (58.80)	270 (48.30)	1.00	1.00

*COR* Crude Odds Ratio, *AOR* Adjusted Odds Ratio

N.B. *=*p*-value<0.05, **p-value<0.01, ***p-value<0.001

The odds of developing Anxiety decreased by 38%; (AOR=0.68, 95% CI=0.42 – 0.92); among participants living with relatives when it compared with relatives living at place possible to visit.

The odds of developing anxiety is 3.92; (AOR= 3.92, 95% CI= 1.99 – 7.69); times increased among divorced participants when it compared with single one.

The odds of developing Anxiety is 2.63; (AOR= 2.63, 95% CI=1.06 - 6.56); times higher among participants living with employers when it compared with housemaids who visit their relatives more frequently.

When compared with participants whose parents are living together, the risk of developing anxiety is 2.45; (AOR= 2.45, 95% CI=1.23 – 4.89); times increased among housemaids from divorced parents, 1.98; (AOR=1.98, 95% CI=1.30 – 3.02); times increased among housemaids who lost father/mother, and 1.13 (AOR= 1.13, 95% CI=0.58 – 2.19) times increased among housemaids who lost both parents.

The odds of developing anxiety is 1.59; (AOR= 1.59, 95% CI=1.09 – 2.33) times more among housemaid who have physical violence.

The odds of developing anxiety is 1.72; (AOR= 1.72, 95% CI= 1.07 -2.78) times more among housemaid who have sexual violence when it compared with no sexual violence.

The risk of developing anxiety is 2.03; (AOR= 2.03, 95% CI= 1.18 – 3.50); times increased among housemaids who are using contraceptive than who are not using contraceptive.

However, in this study there is no significant association identified between age, educational status, salary, service year, substance (khat, alcohol, cigarette) use , DSLE, sexual violence (under 15 & life time) and emotional violence.

## Discussion, Conclusion and Recommendations

### Discussion

In this study; the prevalence of depression, prevalence of anxiety, possible factors associated with depression and factors associated with anxiety were assessed; prevalence of depression and anxiety is 27% and 32 % respectively. Also coincidence of depression with anxiety was analyzed. It was found 44.6% mild form of comorbid depression with anxiety, 18.5% moderate form of comorbid depression with anxiety and 8.1% severe forms of depression comorbid with anxiety.

Findings on prevalence of depression and anxiety is supported by different studies done in Kuwait among housemaids; which describes Psychiatric morbidity among housemaids is two to five times higher than the native female population [7–9].

This finding is higher when compared with the study conducted in Florence (22.8 % depression and 17.2% Anxiety) [5]. The possible reason might be that study conducted among women in general population and this study focus on specific group of population (housemaids) with possibly low socioeconomic characteristics.

Prevalence of depression is higher when it compared with the study done in Mozambique (14%) female heads of household [17]. This difference might be because of the tool used to assess depression. They use PHQ-8, which assess each item of depression and value equally for each item as a screening tool. In case of our study we use PHQ-4, which has two gait symptoms of depression and anxiety for each. Also the study participants are in different status at the household; the head and the servant.

Prevalence of depression is higher when it compared with the study conducted among all Ethiopian women (age 15 – 49) was 4.4% [18]. This difference may occur due to risky age group and socioeconomic status of participants for the study problem. Another study conducted in Ethiopian immigrants at Toronto is less (9.8%) when compare with our study [19].

The odds of developing depression 2.62 times increased among divorced participants when it compared with single one. Also the odd of developing anxiety is 3.92 times increased among those groups of participants when it compared with single one. This finding is supported by the study done in Ethiopian women that explain; odds ratio increased for divorced, separated or widowed women than for not-married women (4.05 and 4.24, respectively) [18].

The odd of developing depression is 2.10 times increased among participants whose parents are divorced and the risk of anxiety is 2.45 times increased among them. This finding is supported by study finding Increased risk for major depression and generalized anxiety disorder was associated with parental separation but not parental death and with separation from either mother or father [20]. But it is against finding on parent’s death. In this study, parent lost has positively associated with depression and anxiety (father/mother lost 1.98 times for both anxiety & depression. Also death of both parents is 1.13 times associate with anxiety) [20].

The risk of developing Depression and Anxiety is increased among participants who had physical violence (1.63 times for depression & 1.59 times for anxiety) and sexual violence (1.59 times for depression and 1.72 times for anxiety). This finding supported by the study conducted in Nicaragua. It states that, *‘Women reporting abuse (any form) were 6 times more likely to experience emotional distress’* [21].

The risk of developing depression and anxiety is (2.19 and 2.03 times respectively) increased among contraceptive users when it compared with participants not using contraceptive.

The odds of developing depression decreased by 74%, 75% and 72% among participants at primary, secondary or tertiary level of education respectively, when it compared with participants who are not educated. This finding is supported by the study conducted in Mozambique; it explains increased risk of depression when additional year of education (AOR: 1.06) [17]

The odd of developing Anxiety is decreased by 38% among participants living with relatives. This finding is supported by Mozambique’s study, additional kilometer from home is risky to develop mental distress (AOR: 1.05) [17]. Also other study explain support from immediate superior find the most consistent protective factor (OR 0.56, 99% CI: 0.43–0.72) among other factors [22].

Unlike other studies, in this study service year, salary, under 15 sexual violence, age, substance use, emotional violence and other variables do not appear to have an independent effect on depression or anxiety [17, 18].

### Limitation

This study has limitation on variables temporal relationship and social desirability bias may affect it. Also as a factor employers don’t consider as a factor despite it has impact on the variable.

## Conclusion

The prevalence of depression and anxiety among housemaid found to be higher at Addis Ababa. Its prevalence is more among age group 16 to 20. This study gives an indication of specific health care needs of women working in household and it is significant public health problem that require a great emphasis. This study weighted the data from Addis Ababa, 2018 to succeed in identifying a statistically significant association on depression and anxiety. The following variables; Educational status, parental relationship, divorce, violence (physical and sexual), contraceptive use and daily stressful life events are found statistically significant.

Preventive measures involving recruitment procedures and pre-employment orientation are needed to minimize the impact of factors on mental health.

## Recommendation

Based on findings and conclusions made, the following recommendations were given for responsible organs.

**To Federal Ministry of health and Addis Ababa health bureau**

- ➢ Consider addressing those groups of female mental health need to achieve health for all agenda
- ➢ Provide psychological and social support

**To Ethiopia Ministry of Women, Children and Youth Affairs:**

- ➢ To arrange possible psychosocial support for those groups of population

**For Researchers:**

- ➢ Further scientific efforts should be made to find out other family and employer related factors

## Data Availability

The datasets generated and analyzed during this study are available as supplementary information file

## Abbreviations

AMSH: Amanuel Mental Specialized Hospital
AOR: Adjusted Odds Ratio
CI: Confidence interval
CIDI: Composite International Diagnostic Interview
DSLEMS: Daily Stress Full Life Events Measurement Scale
GAD: Generalized Anxiety Disorder
PHQ–4: Patient Health Questionnaire -4
PI: Principal Investigator
RW CD: : Reader and Writer Compact Disc
SPSS-20: Statistical Package for Social Sciences version 20
St: Saint
WHO: World Health Organization
